# Zoonotic Parasites in Playgrounds in Southern Spain: A One Health Approach

**DOI:** 10.3390/microorganisms11030721

**Published:** 2023-03-10

**Authors:** Laura Lorenzo-Rebenaque, Sandra López-Fernández, Francisco Marco-Jiménez, Laura Montoro-Dasi, Clara Marin, Santiago Vega, Eduardo Martínez-Manzanares, Fernando Fariñas

**Affiliations:** 1 Institute of Biomedical Sciences, Veterinary Faculty, Universidad Cardenal Herrera-CEU, CEU Universities, Calle Santiago Ramón y Cajal 20, Alfara del Patriarca, 45115 Valencia, Spain; 2 Department Medical Microbiology, Faculty of Medicine, University of Malaga, Calle Louis Pasteur 32, 29010 Malaga, Spain; 3 Institute of Science and Animal Technology, Universitat Politècnica de Valencia, 46022 Valencia, Spain; 4 Institute of Clinical Immunology and Infectious Diseases, Grupo YNMUN Biomedicina, 29018 Malaga, Spain

**Keywords:** *Toxocara*, public health, pets, vector-borne

## Abstract

Zoonotic parasitic diseases are considered a global threat to public health. In this sense, canines and felines may be infected by different cosmopolitan parasites, with playgrounds serving as an important focus of infection for humans, as well as domestic or wild animals. Knowledge of the epidemiological situation of parasites in animal reservoirs integrated into the environment, identifying the spread pathways, is a key element for an effective response to this threat. Thus, the aim of this study was to assess the frequency of intestinal parasites with zoonotic potential in 120 playgrounds in the Malaga province (Spain). Samples were processed and analysed following standard parasitological procedures. Some 36.7% of playgrounds were parasite-positive with one or more zoonotic parasites. The most common parasites recovered were nematodes (60.0%), followed by protozoan species (33.3%) and cestodes (6.7%). In the parasite-positive playgrounds, *Toxocara* spp. (17.0 ± 3.5%) and *Giardia duodenalis* (17.0 ± 3.4%) were the most predominant parasites. In addition, 34.1% of playgrounds were infected with multiple parasites. Our results show a high presence of parasitic forms with zoonotic potential in playgrounds in Malaga, Spain. Due to the close contact between pets and humans in playgrounds, the potential zoonotic risk may increase if prevention and control measures are not designed.

## 1. Introduction

Zoonotic parasitic diseases are deemed a global threat to public health [1,2]. Beyond the high mortality and morbidity rates from parasitic diseases found in developing countries, they also represent a significant human hazard in developed countries that may affect any age group, but have severe consequences in children [1,3]. The disease burden is usually underestimated, due to its absence or limited presence in operational surveillance systems or the lack of routine stool specimen analysis from persons with diarrhoeal illnesses. In this sense, different zoonotic parasitic diseases derived from pets, such as those caused by helminths, for example hydatidosis; or cutaneous or visceral larva migrans syndromes, which are related to hepatomegaly with occasional cerebral and ocular involvement; or by protozoans, such as cryptosporidiosis or giardiasis, that are related to diarrhoea [4]. Moreover, the higher medical and treatment costs constitute a significant economic burden [1]. 

Dogs and cats play an important role in society, with a psychological and physiological impact; they can be guides for blind people, therapeutic agents and are also security guards, children’s friends, or hunters [5]. Europe has one of the highest rates of pet ownership in the world, with 46% of European households owning pets (90 million households), and it is estimated that there is a total of 92 million domestic dogs and 113 million domestic cats [6]. Of the total number of dogs and cats, 10 and 5%, respectively, belong to Spanish families [6]. However, as with other animal species, dogs and cats remain a source of parasitic infections to humans or other animals. In this sense, the close relationship between humans and pets indicates the need for detailed information on zoonotic parasite epidemiology [7,8]. The manifestation of clinical signs in pets depends on the parasite species or the presence of multiple species, their abundance, and on specific animal factors such as age or immunological status [9]. Diarrhoea, vomiting, intestinal obstruction, anorexia and weight loss are the most common clinical signs of parasitic disease in pets. Nevertheless, this could vary depending on the parasitic species and the number of parasites [10]. Playgrounds represent an important focus of infection for humans, particularly children, and other domestic or wild animals [7,11].

Canines and felines can be infected by different cosmopolitan protozoans and helminths. Infection not only causes damage to their organs, but also poses a risk of infection to humans with whom they coexist [11,12]. Playgrounds are leisure environments, where adults and children share the environment closely with dogs of different origins, leaving these areas exposed to human and animal bacteria and virus, as well as intestinal parasites, through soil contamination by faeces from pets and stray dogs [13,14]. In particular, faeces from infected animals contain parasites that could contaminate the environment, acting as a source of infection for people using the parks, especially children, as well as other pets [11]. Knowing the epidemiological situation of the parasite in animal reservoirs integrated into the environment, identifying their pathways of spread, is a key element for an effective response to this threat [15]. In this context, the aim of this study was to assess the frequency of intestinal parasites with zoonotic potential in playgrounds in Malaga province (Spain).

## 2. Materials and Methods

### 2.1. Study Playgrounds

An analytical cross-sectional observational study was conducted in playgrounds in the province of Malaga, Spain. Malaga is a province of the Autonomous Region of Andalusia in the south of the Iberian Peninsula at the Western end of the Mediterranean Sea. It has a surface area of >7300 km^2^ and a total population of 1660 million inhabitants. Malaga has a warm and dry Mediterranean climate, characterised by low temperature oscillation, with long, dry, warm summers and short, mild winters. Average annual temperatures range between 12.5 °C and 19 °C. 

**Figure 1:**
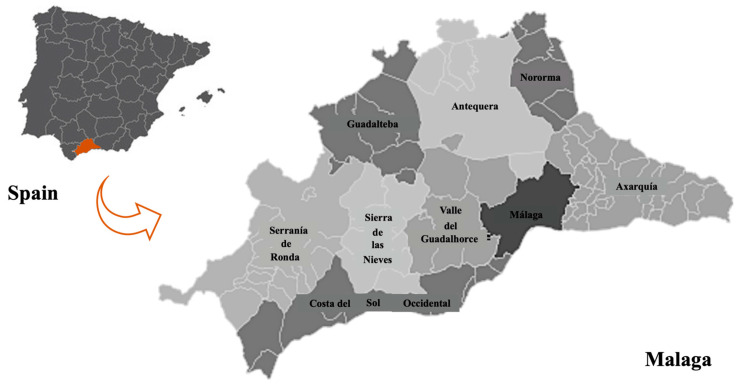
The map of the administrative divisions of the province of Malaga has been enlarged to show the 9 sectors where the sampled playgrounds were located. The localisation of the province of Malaga in Spain is highlighted in orange in the upper left corner.

The study was conducted over 4 months, during which 120 playgrounds were studied in 9 sectors of Malaga: Serranía de Ronda, Costa del Sol Occidental, Sierra de las Nieves, Guadalteba, Valle del Guadalhorce, Malaga, Axarquía, Comarca de Antequera and Nororma (Figure 1).

### 2.2. Sampling

Each playground was sampled once. In total, 240 samples (soil, n = 120 and faecal, n = 120) were collected from 3 different areas of each park from February to March 2020 and from August to November 2020. The samples were placed in sterile plastic containers and analysed within the first 48 h.

### 2.3. Laboratory Diagnosis

The samples were examined as follows. Soil samples were analysed by Baermann funnel migration technique [16] and Ritchie’s method [17]. Meanwhile, faecal samples were analysed by Baermann funnel migration technique [16], Sheather’s flotation technique [17] and the simple sedimentation method [17]. (i) For the Baermann technique, 8–10 g of sample (soil or faecal sample) was placed in the centre of a four-layer gauze, which was closed using a rubber band, and placed in a funnel. The funnel was placed in a stand and had a piece of soft silicone tube attached to the stem, closed with a squeezer clip. The funnel was filled with water at 39 °C and left for 24 h at room temperature. Then, the collected suspension obtained by opening the squeezer clip was centrifuged at 1500× *g* rpm for 1 min and the sediment was then examined using normal saline at 10× and 40× magnification [16]. (ii) For Ritchie’s method, approximately 1 g of soil sample was resuspended in 10 mL of saline solution. The samples were homogenised and filtered through folded gauze with the aid of a funnel. The collected suspension was centrifuged at 2000× *g* rpm for 1 min. Supernatant was removed and the pellet was resuspended with saline solution. The pellet was centrifuged and resuspended until the supernatant looked clear. Then, 10 mL of 10% formaldehyde solution was added to the pellet and left for 10 min at room temperature. After that, 5 mL of ethyl ether was added and the whole solution was shaken vigorously for 30 s. Then, the sample was centrifuged at 1500× *g* rpm for 2 min. Finally, the sediment was examined using normal saline at 10× and 40× magnification [17]. (iii) For Sheather’s flotation technique, approximately 1 g of homogenate sample was mixed with 10 mL of sucrose solution (density 1.18 g/cm^3^). Then, to make a bulging meniscus on each tube, sucrose solution was gradually added while stirring until the top of the tube was reached. Coverslips were placed on the meniscus and left to sit for 5 min, after which a drop of Lugol’s iodine was added before examination under the microscope at 10× and 40× magnifications [17]. (iv) For the simple sedimentation method, 1 g of faeces was mixed with 500 mL of water and passed through a 1 mm pore size wire mesh and the filtrate was passed through a 0.3 mm pore size. After centrifugation at 1500× *g* rpm for 2 min, the pellet was analysed under a microscope at 10× and 40× magnification [17].

Identification and classification were based on the external and internal morphology of egg, oocyst and larval stages, previously described by Ash and Orihel [18]. The egg and oocyst identification was based on visible morphological characteristics, which include the size and shape. The larval identification was based on visible morphological characteristics, which include external body structure, the body length, differences in sex organs, shape of the stoma, and organisation of lips, mouth and tail.

In addition, a molecular technique (real-time PCR) and sequencing were performed to confirm the presence and species of *Toxocara*. To this end, DNA isolations were performed using the DNA extraction kit (Qiamp DNA mini kit, Qiagen, Hilden, Germany) as described by Mikaeili et al. [19]. Then, the nuclear ITS region was amplified using FM1: 5′ TTGAGGGGAAATGGGTGAC 3 and FM2: 5′ TGCTGGAGGCCATATCGT 3 as forward and reverse primers, respectively. For PCR reactions, 25 pmol of each primer, 12.5 μL of PCR premix (AmpliTaq Gold 360 Master Mix, cat. nº. 4398876) and 5 μL of template DNA were used. Amplification was performed with a first denaturation of 12 min at 94 °C, followed by 35 cycles of 30 s at 94 °C, 30 s at 60 °C, 1 min at 72 °C, and final incubation of 5 min at 72 °C. Subsequently, PCR products were run in 1% agarose gel electrophoresis in TBE (Tris 0.09 M, borate 0.09 M, EDTA 0.02 M) at 80 V for 1 h. Gels were stained with Gel Red (GelRed™ nucleic acid gel stain diluted 10,000 times in water, cat. nº. 41003, Biotium, Fremont, CA, USA) and the bands were visualised using a UV transilluminator. 

### 2.4. Statistical Testing

A playground was considered positive if one of the samples (soil or faeces) was positive. A generalised linear model, which assumed a binomial distribution for parasite infection (probit link function), was fitted to the data to determine whether there was an association with the taxonomic category. Data are presented as least squares means ± standard error of the least squares means. A difference of *p* < 0.05 was considered statistically significant. Analyses were conducted using a commercially available software application (SPSS 27.0 software package; SPSS Inc., Chicago, IL, USA, 2020).

## 3. Results

A total of 36.7% (44/120) of playgrounds were parasite-positive with at least one zoonotic species. The lowest proportion of positive samples was recorded in soil samples (20.8%, 25/120), while faecal samples had the highest number of positives (27.5%, 33/120). A total of 60 types of parasites, belonging to 5 parasite species, were detected. The most common parasites recovered from all samples were nematodes, followed by protozoan species and cestodes. Specifically, the parasite species were recovered with the following frequency of occurrence: *Ancylostoma caninum* (6.7%, 4/60), *Toxocara* spp. (35.0%, 21/60), *Uncinaria stenocephala* (18.3%, 11/60), *Giardia duodenalis* (33.3%, 20/60) and *Dipylidium caninum* (6.7%, 4/60).

Regarding the parasite-positive playgrounds (44/120), *Toxocara* spp. and *Giardia duodenalis* were the most predominant, followed by *Uncinaria stenocephala*, *Ancylostoma caninum* and *Dipylidium caninum* (Table 1).

Of the *Toxocara* spp. recovered, 3 playgrounds were infected with multiple parasites (*Toxocara canis* and *Toxocara cati*), while 17 playgrounds were infected with *Toxocara canis* alone. Note that one species of *Toxocara* could not be identified.

Twenty-nine playgrounds (65.9%) were parasitised by one parasite species, while fifteen were infected with multiple parasites. Specifically, 31.8% of the playgrounds were infected with two species (Table 2), while 2.3% (1/44) were infected with three. The frequency of positive samples categorised as co-infections were for *Toxocara* canis together with *Giardia duodenalis* (33.0%, Table 2).

## 4. Discussion

Research into zoonotic parasitic diseases requires a One Health approach, as they are associated with livestock and domestic pets, and their human-to-animal transmission is becoming an emerging public health issue [1]. Animals infected by zoonotic parasites can transmit them through faeces and contaminate the environment [13,20]. Direct or indirect contact with infective parasitic stages from faecal contamination results in human infection, representing a public health hazard [13,20].

In the current study, the findings demonstrated high parasite presence in parasitic forms with zoonotic potential among playgrounds in the province of Malaga, Spain. To our knowledge, the present study provides the first description of the diversity and frequency of zoonotic parasite species in playgrounds in Malaga, Spain.

It should be noted that our results corroborate previous studies performed in other regions of Spain, which show a high prevalence of parasites in public playgrounds. Specifically, rats trapped in parks in Barcelona were positive for zoonotic intestinal protozoans (53%), with *Giardia* spp. (20%) as the most prevalent parasites [15]. Related to this, both urban and rural dogs in Castellón (Mediterranean coast) were positive for intestinal parasites (65.8%), with *Giardia duodenalis* (35.4%) as the most prevalent parasite. In addition, in Valencia, 14 public parks in the city were analysed, and 10.9% of positive soil samples were identified from five parks (35.7% were positive for the presence of *Toxocara* eggs). However, no *Toxocara* species were isolated from faecal samples collected from the parks [21]. Cities promote the transmission of biological agents by creating specific ecosystems with higher temperatures, “urban heat islands” [22]. This fact, coupled with the movement of people and animals in and out of cities, favours indirect zoonotic events that might previously have been limited to remote rural areas but now present greater risks of spreading within cities, which have become a focus of disease transmission, including zoonotic parasite diseases [22]. Playgrounds are meeting places for families and animals, not only pets but also strays. The presence of zoonotic endoparasites in stray animals constitutes a serious risk, as they can contaminate public areas of playgrounds, with children being particularly susceptible [22]. Moreover, feeding raw meat diets to pets also poses a potential risk of transmission of zoonotic parasites such as *Toxoplasma, Neospora, Sarcocystis, Crytosporidium*, *Trichinella* or *Echinococcus* [23]. In this regard, some parasites commonly found in rural areas in food-producing animals may have the opportunity to complete their life cycle, as the relationship between definitive and intermediate hosts becomes closer [23]. Potentially, infected domestic animals can act as definitive hosts, continuously disseminating parasites into the environment, both in the home and on the streets and playgrounds. Therefore owners should adopt hygienic measures to limit infection and disease burden in the household, including routine coprological examinations of pets [23].

In our study, seven parasite species were found, and were consistent with parasite species reported in Spain [15,24]. Indeed, is important to note that the parasite species isolated in this study are widely known as potential zoonotic agents. For instance, *Toxocara* spp. and *Giardia* spp. were the most frequent species found. *Toxocara* spp. are common parasites in dogs worldwide, with zoonotic distributions not only associated with poor knowledge of sanitary practices in developing countries, but also in developed countries with adequate sanitary facilities [20,24]. In this sense, the most important parasites in pets under a One Health paradigm were the intestinal helminths [25]. The ability of *Toxocara* spp. eggs to survive for years in the environment facilitates their transmission [20,22]. Humans become accidental hosts when they ingest dirt contaminated with faeces from animals carrying *Toxocara* infective eggs, such as in playgrounds [26]. However, transmission to humans can also occur through direct contact with pets due to the presence of *Toxocara* infective eggs in pet hair [26]. Although the number of infective eggs in pet hair is very low, it is lowest in well-cared-for, owned adult dogs and highest in puppies and strays [26]. It is important to note that only embryonated eggs are infective, so the environment is an important source of infection [26]. The first human infection was reported in 1950, and it is currently considered that 19.0% of the population have antibodies [27,28]. In Spain, there are no data since 2006, when a study revealed a seroprevalence of 28% in the population [29]. Although progress in treatment protocols has improved control of the disease [30], it should be emphasised that it is necessary to continue improving the protocols and verify their correct application and effectiveness [24]. Likewise, *Giardia duodenalis,* which was first identified in 1681 by Antony van Leeuwenhoek, is the most common human intestinal parasite [31]. Specifically, approximately 280 million human diarrhoea cases are caused by this parasite each year, being particularly significant in children [31]. Moreover, in pets *Giardia duodenalis* causes both symptomatic and asymptomatic infections [32]. Through defecation and spreading of faecal matter, populations of infected dogs can contaminate different environments [33]. Although its zoonotic transmission from pet animals has been described as a rare event, *Giardia* can be transmitted directly from infected domestic or wild animals [32,34,35]. In humans, the infection could be asymptomatic, or cause clinical symptoms ranging from mild diarrhoea to severe malabsorption [35]. In the present study, 15% of playgrounds were positive for *Giardia duodenalis*, in line with results from dog faecal samples in New York City public parks [36] and household dog and cat faeces in Slovakia [37]. However, its frequency may be underestimated, as it may not be detected in a sample if the parasite concentration is low, or the parasite is hidden due to intermittent moulting and bile pigments [35]. These findings suggest that correct deworming protocols are not being implemented and that the risk of infection by zoonotic parasites in public parks and play areas is being underestimated.

In this work, other species were detected, such as *Uncinaria stenocephala*, *Ancylostoma caninum* and *Dipylidium caninum*. It is worth mentioning that these species are also considered the most common contaminants of urban areas, representing a threat to human health which should therefore not be underestimated, especially for children who share playgrounds with pets [13,38]. The third most prevalent species found was *Uncinaria stenocephala*. Note that the larvae of this parasite can survive in the environment for several months, constituting a potential epidemiological hazard [25]. *Uncinaria stenocephala* is zoonotically important and responsible for causing larva migrans syndrome, due to contact with infecting larvae or eggs [39]. This disease has also been linked with *Ancylostoma caninum*. *Ancylostoma* was found in the 3.0% of the playgrounds, which is similar to the results described in dog samples in Slovakia, Serbia and Argentina [38]. Unlike *Uncinaria*, the larvae of this parasite could survive in the environment for 3–4 weeks [25]. In this sense, warm and humid environments are necessary for *Ancylostoma* larvae development, so this parasite is usually more prevalent in a sylvatic context than in urban areas [25]. Finally, *Dipylidium* has usually been considered the most frequent cestode in dogs [39]. *Dipylidium* is transmitted by intermediate arthropod hosts; thus, its presence its related not only to infected dogs, but also to the vectors of this tapeworm, i.e., fleas [13]. Although direct transmission from animal to human is rare, by the inadvertent ingestion of cysticercoids (i.e., sucking hands after petting an infected animal), it is important to highlight that rigorous faecal removal practices should be encouraged to limit their presence in the environment and their possible transmission through vectors.

Moreover, most samples were infected with only one parasite species, in line with previous studies [40]. However, a high percentage of samples were infected by multiple parasite species (39%), probably due to ecological and environmental associations [41]. This finding is of general interest, as it could increase the susceptibility to other infections, with an impact on morbidity, immune reaction to the treatment and reinfection rates [41]. To address this problem, more epidemiological studies under the One Health paradigm, using a multidisciplinary approach, are essential [38]. Moreover, there is a need to engage in more in-depth veterinary control of owned dogs, including routine coprological and diagnostic methods for the rapid detection of gastrointestinal parasite infections in domestic animals, while toughening municipal laws on faeces collection and improving the owners’ knowledge of the risks of these infections [13,38].

## 5. Conclusions

Our results show a high presence of parasitic forms with zoonotic potential in playgrounds in Malaga, Spain. Due to the close contact between pets and humans in playgrounds, the potential zoonotic risk may increase if prevention and control measures are not designed. 

## Figures and Tables

**Table 1:** Prevalence of playground zoonotic parasites.

Phylum	Order	Family	Genus	Species	n	Total (%)
Nematoda	Strongylida	Ancylostomatidae	*Ancylostoma*	*A. caninum*	4	3.0 ± 1.6 ^b^
	Ascaridida	Ascarididae	*Toxocara*	*Toxocara* spp.	21	17.0 ± 3.5 ^a^
	Rhabditida	Ancylostomatidae	*Uncinaria*	*U. stenocephala*	11	9.0 ± 2.6 ^ab^
Sarcomastigophora	Diplomonadida	Hexamitidae	*Giardia*	*G. duodenalis*	20	17.0 ± 3.4 ^a^
Platyhelminthes	Cyclophyllidea	Dilepidiidae	*Dipylidium*	*D. caninum*	4	3.0 ± 1.6 ^b^

n = Total number of samples positive for respective parasite across the 120 playgrounds; % = Percentage of positive samples of the total number of playgrounds. ^a–b^: Different superscripts letters in the same column indicate significant differences (*p* < 0.05). Data are presented as least squares means ± standard error of the least squares means.

**Table 2:** Co-infections of playground zoonotic parasites.

Parasites			n	Total (%)
*Toxocara canis*	*Giardia duodenalis*		5	33.0
*Toxocara canis*	*Uncinaria stenocephala*		3	20.0
*Giardia duodenalis*	*Uncinaria stenocephala*		2	13.0
*Toxocara canis*	*Ancylostoma caninum*		1	7.0
*Uncinaria stenocephala*	*Dipylidium caninum*		1	7.0
*Uncinaria stenocephala*	*Ancylostoma caninum*		1	7.0
*Giardia duodenalis*	*Dipylidium caninum*	*Uncinaria stenocephala*	1	7.0
*Toxocara canis*	*Toxocara catis*	*Dipylidium caninum*	1	7.0

n = Total number of samples positive co-infected across the 120 playgrounds; % = Percentage of positive samples co-infected of the total number of playgrounds.

## Data Availability

Not applicable.
